# Reduction of Friction and Wear for AISI321 Stainless Steel through Surface Modification Using Nanocrystallization

**DOI:** 10.3390/ma16155303

**Published:** 2023-07-28

**Authors:** Lifen Ding, You Li

**Affiliations:** 1 School of Mechanical and Electronic Control Engineering, Beijing Jiaotong University, Beijing 100044, China; 2 Guangzhou Locomotive Depot of Guangzhou Shenzhen Railway Co., Ltd., Shenzhen 510010, China

**Keywords:** nanocrystallization, sulfurization, tribological properties, physical model, compound-modified layer

## Abstract

Through surface nanocrystallization and low-temperature ion sulfurization, the nanocrystalline/FeS thin film with excellent friction-reduction and antiwear properties was fabricated on the surface of AISI321 stainless steel. The nanocrystallization treatment formed the high hardness and active nanocrystalline structure on the surface of AISI321, with the harness increased from 4.6 GPa to 7.56 GPa. Furthermore, the significantly refined nanostructure strongly increased the concentration of S element in comparison with the single-sulfurized layer on the substrate. Tribological tests reveal that both the original AISI321 substrate and the single-sulfurizing-treated samples are subject to severe abrasion. Single nanocrystallization treatment can improve the wear resistance of AISI321, while the compound treatment can obviously improve the comprehensive tribological properties. The compound-modified layer presents excellent tribological properties with the lowest coefficient of friction (COF) of 0.33, which is related to the increased hardness of the substrate and increased thickness, density, and homogeneity of the sulfurized layer. Furthermore, a physical model is developed for the vacuum tribological behavior of the samples after different treatments. This model provides a reference for revealing the tribological mechanism of the compound-modified layer treated using surface nanocrystallization-assisted chemical heat treatment.

## 1. Introduction

Due to its good corrosion resistance, creep strength, tensile strength, and high-temperature mechanical properties, AISI321 austenitic stainless steel is wildly used in the manufacture of mechanical components, such as bearings, pump valves, and medical devices [1,2]. However, the relative low surface hardness, poor wear resistance, and easy adhesive wear performance limit its service life [3]. Therefore, surface modification technologies are necessary to be applied for improving its friction-reduction and antiwear properties [4,5,6].

As one of the chemical heat treatment processes, sulfurization can realize the infiltration of S atoms into steel and form a sulfide solid lubricating film on FeS-based solid lubricating film with a few microns of thickness. Herein, as the friction coefficient can be effectively reduced and the antiscuffing performance can be improved. Sulfurization plays an important role in enhancing the tribological properties of mechanical parts and extending their service life [3,7]. However, during the service, the low hardness of the sulfurized layer causes the deterioration of sulfides. Thus, in order to achieve the double improvement in friction-reduction and antiwear properties, the sulfurization treatment is often combined with a surface strengthening treatment [8,9,10].

Nanocrystallization of metal surfaces is an advanced surface engineering nanotechnology, which can strongly increase surface activity without changing the chemical composition of the substrate material [11,12,13]. In particular, the developed supersonic fine particles bombarding (SFPB) technology can produce a uniform nanocrystallized layer on the metal surface regardless of the size and shape of the workpiece [14,15]. As the surface physical and chemical properties [14,16], strength and hardness [17] and tribological properties [17] of metallic materials can be highly improved, SFPB has a wide range of applications in the field of mechanical engineering.

In this work, to obtain the compound-modified layer (nanocrystalline/FeS film) with excellent tribological properties, AISI321 stainless steel was treated using SFPB-assisted sulfurization [18]. The macro–micro friction-reduction and antiwear mechanisms of the compound-modified layer were systematically investigated. Finally, a physical model regarding the tribological behavior of the compound-modified layer was proposed.

## 2. Experimental

The substrate of AISI321 stainless steel was treated with solid solution of 1100 °C × 2 h. The chemical components of AISI321 stainless steel (wt%) are given in Table 1.

All samples were divided into 4 groups: the as-prepared AISI321 steel sample (AP-ed); the surface nanocrystallization treated sample (SN-ed); the sulfurizing treated sample (ST-ed); the surface nanocrystallization- and sulfurizing-treated sample (SNT-ed). SFPB technology was used for surface nanocrystallization; more details can be found in reference [9]. The parameters of SFPB were summarized in Table 2. Low-temperature ion sulfurization technology was used for sulfurization, and the process details can be seen in reference [10]. In this work, the sulfurizing temperature and time were designed as 220 °C and 1.5 h, respectively. During cooling, the nitrogen was charged into the furnace. Then, the sample was taken out and vacuum-packed.

A scanning electron microscope (SEM, FEI Quanta 200) and transmission electron microscope (TEM, JEM-2010) were used to analyze the surface morphology before and after SFPB treatment. A Field emission scanning electron microscope (FESEM, JSM-6301F) and auger electron spectroscopy (AES, JSM-6301F) were used to detect surface morphology and element (Fe and S) distribution in the depth direction of ST-ed and SNT-ed. The nanoindentation instrument (Nano-600) was adopted to measure the nanohardness and elastic modulus of all samples. The penetration depth is fixed at 150 nm.

The tribological tests were carried out using the ball-on-disk-type tribometer (УTИ-1000) under the environment of 1 × 10^−4^ Pa. Wear tests were carried out with loads of 8 N, a sliding velocity of 0.4 m/s, and wear time of 20 min. The counterbody comprises 9Cri18 balls with a high microhardness of HRC58, a diameter of 9.525 mm, and a surface roughness of Ra 0.032 μm. The morphology of the wear surface was further analyzed using a confocal laser scanning microscope (CLSM) and SEM.

## 3. Results and Discussion

### 3.1. Characterization of Nanostructured Layer on AISI321 Stainless Steel Treated Using SFPB

**Figure 1:**
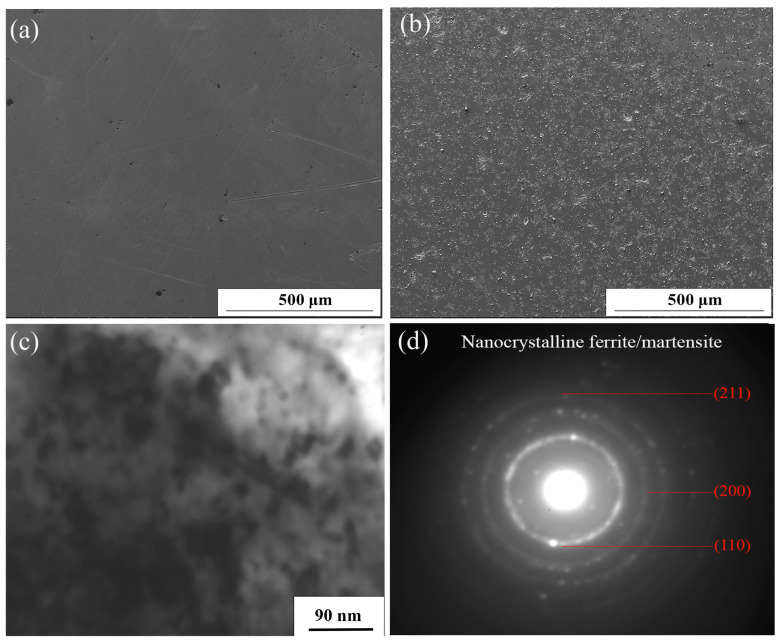
SEM and TEM morphologies of AISI321 stainless steel surface before and after SFPB: (**a**) Surface morphology of AP-ed; (**b**) Surface morphology of SN-ed; (**c**) Nanocrystallized layer; (**d**) Selected area diffraction pattern of the nanocrystallized layer.

Figure 1 shows the SEM and TEM morphology of the AISI321 surface before and after SFPB nanocrystallization treatment. From Figure 1a,b, due to the forward extrusion effect of high-energy particles, nonuniform plastic deformation happened on the AISI321 surface. A large number of small pits appeared, which strongly increases the surface roughness. Furthermore, the SN-ed surface was analyzed using TEM and the results are shown in Figure 1c,d. After SFPB, the grains of AISI321 surface were refined into equiaxial nanograins of different sizes, with about 21 nm and random orientation. As shown in Figure 1d, the diffraction rings appeared in the selected area electron diffraction (SAED), which confirms that the nanograins of AISI321 formed. Therefore, SFPB can strongly refine the surface microstructure of AISI321.

### 3.2. Effect of SFPB on the Microstructure and Properties of Sulfurized Layer

#### 3.2.1. Effect of SFPB on the Microstructure of Sulfurized layer

**Figure 2:**
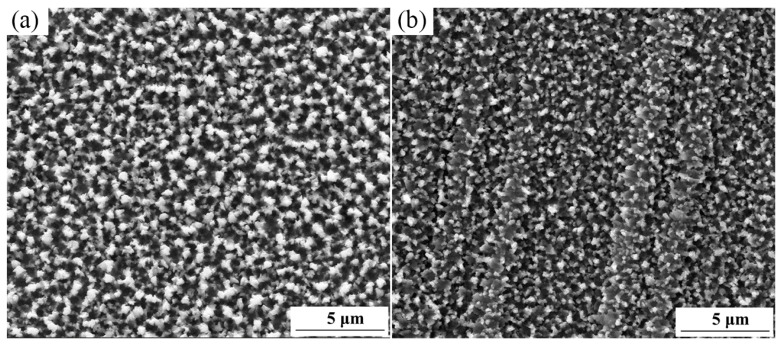
Surface morphologies of the sulfurized layer before and after SFPB: (**a**) ST-ed layer; (**b**) SNT-ed layer.

Figure 2 shows the surface morphology of the sulfurized layer before and after SFPB. It is clear that the SFPB can promote the nucleation of sulfides and refine its grain size. After SFPB, the average grain size of the sulfurized layer was reduced from 600 μm to 100 μm.

**Figure 3:**
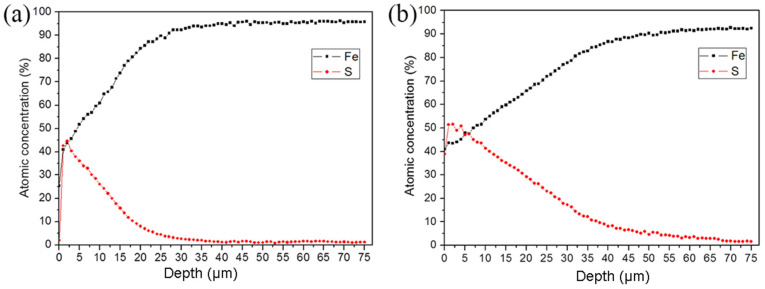
AES analysis of Fe and S element distribution along the depth direction after sulfurization treatment with a sputter velocity of 50 nm/min: (**a**) ST-ed layer; (**b**) SNT-ed layer.

Figure 3 shows the distribution of the main elements (Fe and S) of the sulfurized layer in the ST-ed and SNT-ed samples along the depth direction. The variation in Fe and S element content shows the opposite trends along the depth direction in both ST-ed and SNT-ed layers. In detail, the S element content of SNT-ed reaches 52 at.%, which is higher than that of the ST-ed sample with one maximum content of S element 45 at.%. At the same time, the infiltration thickness is much larger in the SNT-ed sample. Even at the depth of 60μm, the S element (~5 at.%) can still be detected. Differently, for the ST-ed, the content is less than 5 at.% at the depth of 30 μm. Therefore, SFPB plays an important role in promoting S element diffusion and enlarging the S element concentration and thickness of the sulfurized layer.

#### 3.2.2. Effect of SFPB on the Mechanical and Tribological Properties of Sulfurized Layer

**Figure 4:**
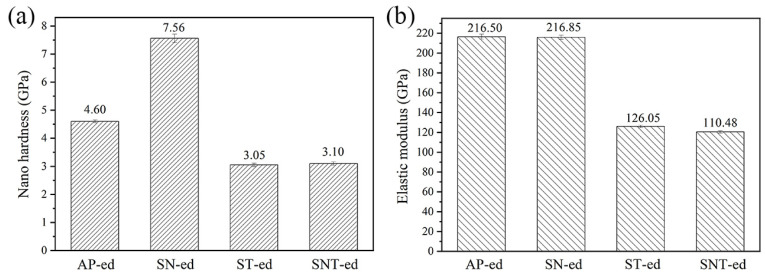
Comparisons of nanomechanical properties of samples after different treatments: (**a**) Nanohardness; (**b**) Elastic modulus.

Figure 4 compares the hardness and elastic modulus of different samples measured using nanoindentaion. After AFPB, the hardness increased from the 4.6 GPa of the AP-ed sample to be 7.56 GPa of the SN-ed sample. Numerous studies have confirmed that the formation of nanograins and dislocations after nanocrystallization are important factors in improving surface hardness [18,19,20]. Therefore, the formation of nanograins after SFPB plays the fine grain strengthening effect on AISI321 stainless steel. After sulfurization, both hardness values decreased to be 3.05 GPa and 3.10 GPa for ST-ed and SNT-ed samples. This is resulted from the low hardness of the sulfide phase formed on the surface of AISI321 stainless steel. Compared to the ST-ed, SNT-ed has a finer and more uniform microstructure. Thus, the nanohardness of SNT-ed is slightly higher than the ST-ed. Figure 4b shows that SFPB has no effect on the elastic modulus of AISI321 stainless steel. However, after sulfurization, the elastic modulus of ST-ed and SNT-ed samples obviously decreased. It indicates the formation of finer sulfides and reduces the interatomic bonding force in the sulfurized layer.

**Figure 5:**
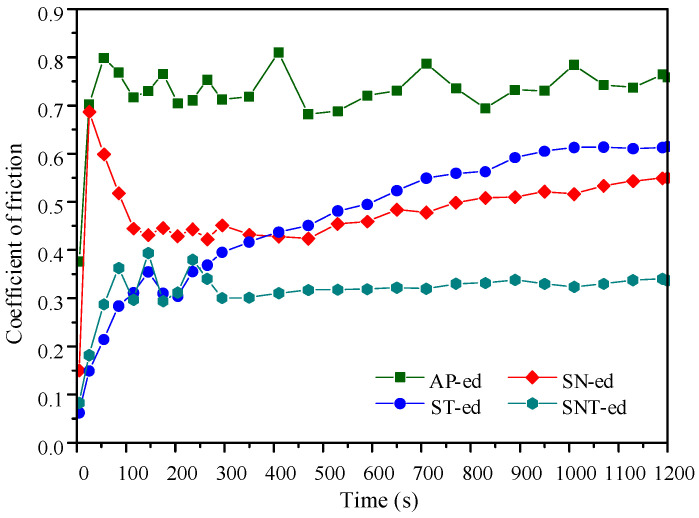
The variation in coefficient of friction with time in different samples.

Figure 5 shows the coefficient of friction (COF) curves of the four categories samples. After the running-in stage with one period of 150 s, all samples transformed into the steady-wear stage except the ST-ed sample. It is clear that the COF of the differently treated samples is lower than AP-ed. Especially, the SNT-ed layer presents the lowest COF (0.33). This value is 40% and 50% lower than the SN-ed and ST-ed layers, respectively. This demonstrates that the combination of nanocrystallization and sulfurization treatments can strongly optimize the surface performance. The ST-ed layer has a lower COF in the running-in stage due to the formation of the FeS phase with strong plastic rheological capacity. However, the ST-ed layer loses the lasting antiwear capacity in a later stage due to the thin sulfurized layer and low wear resistance. It can be concluded that the SNT-ed layer has the best friction-reduction property combined with the merits of both the high wear resistance of the nanocrystallized layer and the excellent friction-reduction property of the sulfurized layer.

**Figure 6:**
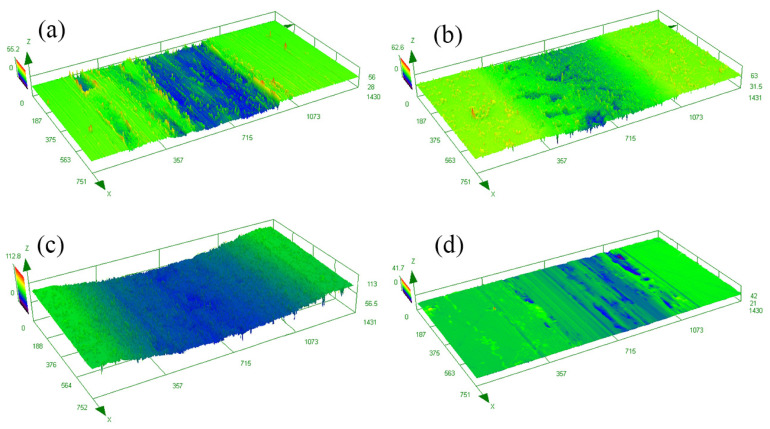
3D morphologies of the wear tracks of the samples after different treatments: (**a**) AP-ed; (**b**) SN-ed; (**c**) ST-ed; (**d**) SNT-ed.

Figure 6 reveals the three-dimensional (3D) morphology of the wear tracks of the samples after different treatments. The severe abrasion appeared on the surface of AP-ed and ST-ed layers, especially on the ST-ed layer with the largest wear width and depth. In contrast, only minor pits and slight wear tracks existed on the surface of SN-ed and SNT-ed layers. Moreover, the ratio between hardness/elastic modulus (H/E) value was calculated using the nanoindentation test, which is positively correlated with the wear resistance of the sample during the wear process [21]. H/E values of AP-ed, SN-ed, ST-ed, and SNT-ed layers are 0.021, 0.035, 0.024, and 0.028, respectively. Due to the SFPB treatment, SN-ed and SNT-ed layers have higher H/E values. Herein, their wear resistances are better than ST-ed layer. During the wear process, the formation of hard-abrasive particles of SN-ed and SNT-ed layers causes less damage. Based on the wear width and depth, the order of wear loss from large to small is the ST-ed layer, AP-ed layer, SN-ed layer, and SNT-ed layer. Among them, the ST-ed layer has the most serious wear loss due to the lowest nanohardness. Although the AP-ed layer has a higher nanohardness, it still exhibits larger wear loss because of the poor antiwear capacity. The SN-ed layer presents a good friction-reduction property, but its wear loss is still worse than that of the SNT-ed layer due to the lack of antiwear lubricating phase.

#### 3.2.3. Friction-Reduction Mechanism Model of Compound-Treated Layer

**Figure 7:**
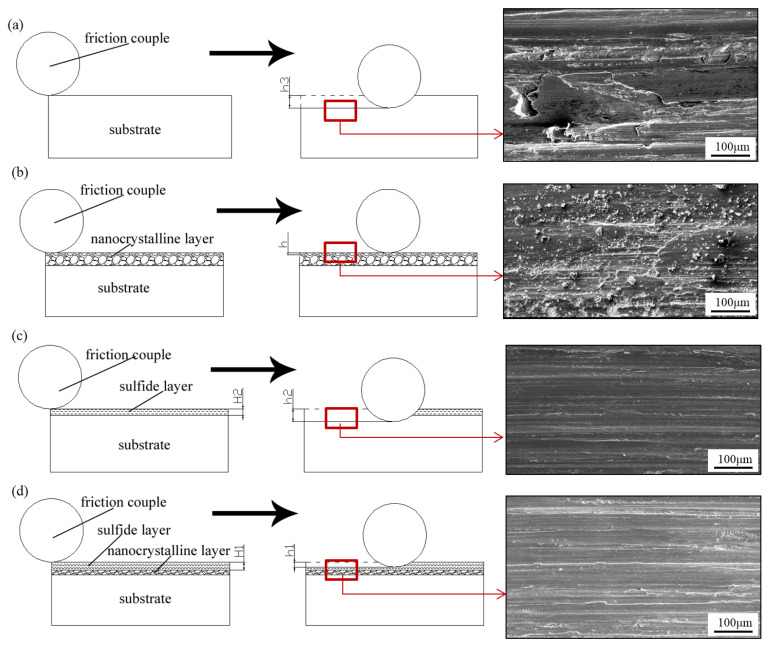
Physical models of the wear process of the samples after different treatments: (**a**) AP-ed; (**b**) SN-ed; (**c**) ST-ed; (**d**) SNT-ed. The areas marked by red squares are observed by SEM imges.

The above experimental results show that the friction and wear properties of AISI321 stainless steel are greatly improved after the combined treatments. As shown in Figure 5 and Figure 6, sulfurization treatment strongly reduces the COF of AISI321 stainless steel, while the surface nanocrystallization treatment improves its wear resistance. Based on the above analysis, as shown in Figure 7, the physical model regarding friction reduction and antiwear was proposed for the modified layers. The left images represent the moment when the friction pair has just contacted but not yet worn out, the middle images represent the moment when the friction pair has slid a certain distance, reaching a certain level of abrasion damage, and the right images are the corresponding SEM morphologies of the red enlarged zones. Figure 7a reveals that serious plastic deformation including ploughing grooves and adhesive tearing happened in the surface of AP-ed. As the hardness of AISI321 stainless steel is lower than that of 9Cri18 steel, a 9Cri18 ball can easily penetrate into the soft AISI321 substrate in the early sliding stage. The surface of AP-ed was gradually pushed by the friction ball under tangential stress. At the contact peak position between the friction ball and substrate, longitudinal stress exceeds the ultimate compressive yield stress of the substrate, leading to strong plastic deformation. Therefore, the substrate occurred severe wear and a deeper wear track (h3). However, due to the nonuniform distribution of contact stress, partial adhesion and tearing from substrate occurred, forming wear tracks with different depths. Differently, Figure 7b shows that the wear tracks of the SN-ed layer are much smaller than that of AP-ed. No adhesion and tearing happened, while more small wear debris appeared on the surface. The formation of nanograins treated using SFPB contributes to the increase in hardness. It leads to the reduced depth of abrasive grain pressing into the nanocrystallized layer and the contact area of the friction interface. According to the friction binomial law [22], less mechanical engagement and molecular attraction of the contact interface lead to a lower COF. The hard asperity decreases the cutting and adhesion effects. The weakened cutting and adhesion effects of hard asperity on materials lead to shallower wear tracks (h_0_ ≈ 0). However, the increased surface roughness causes the formation of more abrasive grains and less uniform wear.

After sulfurization, Figure 7c shows that only slight adhesive wear occurs on the surface of ST-ed layer. The formation of FeS film avoids the direct contact between the friction pair and substrate. The dense hexagonal crystal structure of FeS film is prone to slip along the close-packed plane and exhibits strong plastic rheological capacity, which avoids the occurrence of adhesion tearing. Nevertheless, the lower hardness of the sulfurized layer leads to lower hardness of its underlying substrate. Under positive pressure, The sulfurized layers contribute to provide effective support for FeS film, and then undergoes strong plastic deformation with collapse and destruction. Hard asperity pierces through FeS film and causes direct contact between metals. Thus, the ST-ed layer shows a low COF in the early wear stage. But, in the later wear stage, its COF rises rapidly too close to the value of the substrate, generating higher wear loss and deeper wear tracks (h_2_ > h_3_). Similar to the ST-ed, Figure 7d shows that numerous ploughing grooves appear on the surface of the SNT-ed layer. However, its wear depth is more shallow than that of the ST-ed layer. The FeS film and nanocrystallized layer play a role in lubrication and antiwear, respectively. Compared to the thickness and hardness of the sulfurized layer and nanocrystallized layer, surface nanocrystallization-assisted sulfurization treatment can form the thicker (h_1_ > h_2_), denser, and more uniform FeS film, improving the antiwear effect of the sulfurized layer. Furthermore, this compound treatment generates the ideal tribological surface with internal hard and external soft, which results in the lowest wear loss and shallower wear tracks (h_1_ < h_2_ < h_3_).

## 4. Conclusions

The nanocrystalline FeS thin film was successfully fabricated on the surface of AISI321 stainless steel through SFPB and low-temperature ion sulfurization. The compound-treated SNT-ed layer shows the lowest COF (0.33) and persistent lubrication effect, which exhibits excellent friction-reduction and antiwear properties. The SNT-ed layer generates the composite surface layer with internal hard and external soft on the sample. With the support of lubricated FeS film and a high-hardness nanocrystallized layer, SNT-ed shows a good wear-resistant capacity and resists the cutting effect of the hard asperity on friction pair under positive pressure. Our developed technique can be industrialized in the manufacture of mechanical components for improving their antiwear and anticorrosion performances, such as bearings, pump valves, and medical devices.

## Figures and Tables

**Table 1:** Process chemical components of AISI321 in wt%.

C	Si	Mn	S	P	Cr	Ni	Ti	Fe
0.1	1.0	1.0	0.003	0.035	17.5	8.5	0.4	Bal.

**Table 2:** Process parameters for surface nanocrystallization using SFPB.

Airflow Pressure	Jetting Medium	Particle Diameter	Bombardment Distance	Bombardment Angle	Processing Time	Airflow Temperature
1.6 MPa	Al_2_O_3_ powder	20~30 μm	15 mm	90°	360 s	50 °C

## Data Availability

Not applicable.
